# Ratio between negative and positive lymph nodes is a novel prognostic indicator for patients with esophageal cancer: A Surveillance, Epidemiology and End Results database analysis

**DOI:** 10.1111/1759-7714.13688

**Published:** 2020-10-09

**Authors:** Wanyi Xiao, Huagang Liang, Hongdian Zhang, Ran Jia, Yueyang Yang, Yang Wang, Peng Tang, Zhentao Yu

**Affiliations:** ^1^ Department of Esophageal Cancer, Key Laboratory of Cancer Prevention and Therapy of Tianjin, Tianjin's Clinical Research Center for Cancer, National Clinical Research Center of Cancer Tianjin Medical University Cancer Institute and Hospital Tianjin China; ^2^ Department of Thoracic Surgery The First Hospital of Qinhuangdao Qinhuangdao China; ^3^ Department of Immunology Tianjin Medical University Cancer Institute and Hospital Tianjin China

**Keywords:** Esophageal cancer, prognosis, the ratio between negative and positive lymph nodes, tumor‐R_NP_‐metastasis stage

## Abstract

**Background:**

The aim of this study was to explore whether the ratio between negative and positive lymph nodes (R_NP_) could predict the overall survival (OS) of esophageal cancer (EC) patients with lymph node metastasis following esophagectomy.

**Methods:**

We utilized the Surveillance, Epidemiology and End Results (SEER) database to include the records of 2374 patients with lymph node metastases post‐surgery. All patients were randomly assigned into the training cohort (*n* = 1424) and validation cohort (*n* = 950). Multivariate Cox regression analyses were performed to identify independent prognostic factors. A novel R_NP_ ‐based TR_NP_M staging system was proposed. The prognostic value of N, R_NP_, TNM and TR_NP_M staging system was evaluated using the linear trend χ^2^ test, likelihood ratio χ^2^ test, and Akaike information criterion (AIC) to determine the potential superiorities. We constructed nomograms to predict survival in both cohorts, and the calibration curves confirmed the predictive ability.

**Results:**

Univariate analyses showed that N and R_NP_ stage significantly influenced the OS of patients. Multivariate analyses revealed that R_NP_ was an independent prognostic predictor in both the training and validation cohorts. For the stratification analysis in the two cohorts, we found significant differences in the prognosis of patients in different R_NP_ groups on the basis of the different N stages and the number of dissected lymph nodes. In addition, the lower AIC value of R_NP_ stage and TR_NP_M staging system represented superior predictive accuracy for OS than the N stage and TNM staging system, respectively. Furthermore, the calibration curves for the probability of three‐ and five‐year survival showed good consistency between nomogram predictive abilities and actual observation.

**Conclusions:**

We demonstrated that compared to the classical pathological lymph nodal staging system, the R_NP_ stage showed superior predictive accuracy for OS and can serve as a more effective prognostic guidance for lymph node positive EC patients.

## Graphical abstract

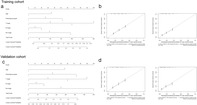

## Introduction

Esophageal cancer (EC) is a highly invasive digestive system malignancy characterized by rapid growth and early metastasis.^1^ Esophagectomy with lymphadenectomy has been applied as the standard treatment modality for potentially resectable EC. However, despite significant progress in multimodal treatment in recent years, the prognosis for patients with EC remains poor.^2^, ^3^

The identification of prognostic factors for EC is extremely important in predicting prognosis and guiding treatment. The postoperative pathological lymph node (N) staging is a basic staging of lymph node metastasis in line with the eighth edition of the American Joint Commission on Cancer (AJCC) criteria.^4^ However, the number of metastatic lymph nodes depends on the number of dissected lymph nodes. A low number of examined lymph nodes may lead to stage migration.^5^ To improve the existing prognostic evaluation system, we aimed to identify the optimal prognostic indicators for EC patients. The number of negative lymph nodes (NLNs) is the difference between the total number of completely removed lymph nodes (RLNs) and the number of positive lymph nodes (PLNs). Previous studies have shown that the NLN count is a valuable predictor of prognosis in various cancers.^6^, ^7^, ^8^, ^9^, ^10^ Several studies have also demonstrated that the number of NLNs is positively correlated with the OS of EC patients.^11^ The higher the number of NLNs a patient has, the better the prognosis.^12^, ^13^

The ratio of NLNs to PLNs (R_NP_) is obtained by taking the ratio of the number of NLNs to the number of PLNs. Several studies have validated that the R_NP_ is a novel prognostic predictor in colon cancer and gastric cancer patients post‐surgery.^14^, ^15^, ^16^ However, the prognostic performance of R_NP_ in EC patients is currently unknown.

Therefore, the purpose of this study was to elucidate the value of R_NP_ in predicting the long‐term survival of EC patients using a population‐based analysis of the Surveillance, Epidemiology and End Results (SEER) database.

## Methods

### Study population and data source

Utilizing the SEER database, we performed a retrospective study and analyzed the medical records of 5977 EC patients. Clinical data such as patient demographics, lymph node staging and survival data were collected for subsequent analyses.

The EC incidence data were collected from the SEER database, which is originally sourced from publicly available datasets incorporating data from approximately 29% of the US population. The OS of EC patients post‐esophagectomy in the SEER database were estimated. We compared the OS using univariate Kaplan‐Meier survival analyses and multivariate Cox regression analyses.

The inclusion criteria were as follows: (i) no distant metastasis; (ii) no preoperative neoadjuvant therapy, including chemotherapy, radiotherapy or chemoradiation; (iii) negative incision margins; (iv) confirmed by postoperative histopathological examination; (v) no perioperative death; and (vi) death due to EC progression or cancer‐related complications. The exclusion criteria included: (i) presence of other pathological types except for squamous cell carcinoma and adenocarcinoma; (ii) no positive lymph node metastases; (iii) relevant clinicopathological information was incomplete; and (iv) incomplete follow‐up data.

After screening, a total of 2374 EC patients who met the specified criteria were assigned into the training and validation cohorts by random assignment, with a study endpoint of OS.

### Lymph node classifications

Using the newly published eighth edition of the AJCC staging system, lymph node status was classified using the metastatic lymph node counts as follows: N1, 1–2 regional lymph node metastases; N2, 3–6 regional lymph node metastases; and N3, ≥7 regional lymph node metastases. The tumor‐node‐metastasis (TNM) staging system is as follows: IIB, T1N1M0; IIIA, T1N2M0, T2N1M0; IIIB, T2N2M0, T3N1M0, T3N2M0, T4aN1M0; IVA, T1N3M0, T2N3M0, T3N3M0, T4aN2M0, T4aN3M0. The number of NLNs was obtained by subtracting the number of PLNs from the total number of RLNs. The NLN intervals were as follows: NLN1 (≤7), NLN2 (8–13), and NLN3 (≥14).

The R_NP_ is defined as the ratio of the number of NLNs to the number of PLNs. We performed the following analysis to identify the appropriate cutoff point for the R_NP_ value to maximize the significant survival differences between the various subgroups. We ranked the R_NP_ values and divided the patients into 10 groups in a 10% proportion, compared the five‐year survival rate, and used log‐rank test to combine the neighborhood OS curves to determine the intervals of R_NP_ classification. To match the N stages with the TNM staging system, the patients were divided into three subgroups: R_NP_1 (R_NP_ ≥ 6.3), R_NP_2 (2.2 ≤ R_NP_ < 6.3), R_NP_3 (0 ≤ R_NP_ < 2.2). Furthermore, to ensure comparability with the TNM staging system, we utilized a novel Tumor‐R_NP_‐Metastasis (TR_NP_M) staging system based on the R_NP_ classification. The TR_NP_M staging system was set‐up by replacing the N stage of the traditional TNM staging system with the matched R_NP_ subgroups. The TR_NP_M staging system is as follows: IIB, T1R_NP_1M0; IIIA, T1R_NP_2M0, T2R_NP_1M0; IIIB, T2R_NP_2M0, T3R_NP_1M0, T3R_NP_2M0, T4aR_NP_1M0; IVA, T1R_NP_3M0, T2R_NP_3M0, T3R_NP_3M0, T4aR_NP_2M0, T4aR_NP_3M0.

### Statistical analysis

We used the Statistical Package of Social Science 26.0 software (IBM Corp., Armonk, NY, USA) for statistical analyses. Kaplan‐Meier curves were used for overall survival analyses, and log‐rank tests were utilized for comparison. As with the multivariate survival analyses, significant prognostic predictors for OS from the univariate analyses were used for Cox regression analyses and the factors that remained statistically significant were identified to be independent factors in the final models of the effect on prognosis. All the curves are depicted using GraphPad Prism 8 (GraphPad Software, LLC). A two‐sided *P*‐value of <0.05 was considered statistically significant.

The nomogram was formulated to provide visualized risk prediction using R project version 4.0.2 (http://www.r-project.org/) with the survival and rms package. The calibration curves were finally derived through regression analysis. The performance of the resulting nomogram was internally and externally validated by calculating the concordance index (C‐index).

The Akaike information criterion (AIC) value within a Cox proportional hazard regression model was calculated to compare performances among different lymph node staging systems because of its discriminatory ability. The lower the AIC value, the better the model for predicting outcome. By contrast, a higher linear trend χ^2^ score or likelihood ratio χ^2^ score verified a better model for predicting outcome.

## Results

### Demographics of patients

The detailed clinicopathological characteristics in the training and validation cohorts are shown in Table 1. A total of 60% of the participants (*n* = 1424) were randomly assigned to a training cohort, whereas the remaining 40% were included in a validation cohort (*n* = 950). For the whole study population, there were 2055 males (86.6%) and 319 females (13.4%). The median age was 64 years old, with a range of 23–92 years old. In the training cohort, there were 776 patients in stage N1, 429 patients in stage N2, and 219 patients in stage N3. For the validation cohort, there were 568 patients in stage N1, 256 patients in stage N2, and 126 patients in stage N3. Based on the number of negative lymph nodes, patients were split up into three groups: NLN1 (*n* = 505), NLN2 (*n* = 361), and NLN3 (*n* = 558) in the training cohort. For the validation cohort, 326, 251 and 373 patients were split up into NLN1, NLN2 and NLN3 groups, respectively. Furthermore, based on the R_NP_ value, there were 554 patients in stage R_NP_1, 411 patients in stage R_NP_2, and 459 patients in stage R_NP_3 in the training cohort, while the number of patients in the validation cohort classified into R_NP_1, R_NP_2, R_NP_3 were 398, 301 and 251, respectively.

**Table 1:** Univariate analysis of prognostic factors influencing the survival of esophageal cancer patients with lymph node metastasis

Variable	Training cohort		*P*‐value	Validation cohort		*P*‐value
	Numbers	HR (95% CI)		Numbers	HR (95% CI)	
Gender, male/female	1226/198	0.904 (0.757–1.081)	0.268	829/121	1.245 (0.992–1.561)	0.058
Age, <65/≥65	744/680	1.270 (1.126–1.432)	**<0.001**	555/395	1.406 (1.205–1.640)	**<0.001**
Tumor location, upper/middle/lower	19/141/1264	0.970 (0.827–1.138)	0.706	16/79/855	0.916 (0.747–1.122)	0.395
Tumor size, <40 mm/≥40 mm	599/825	1.248 (1.104–1.411)	**<0.001**	453/497	1.282 (1.100–1.494)	**0.001**
Histological type, G1/G2/G3	47/496/881	1.289 (1.153–1.441)	**<0.001**	33/392/525	1.450 (1.259–1.670)	**<0.001**
T stage, T1/T2/T3/T4a	158/199/968/99	1.158 (1.115–1.204)	**<0.001**	116/149/632/53	1.136 (1.081–1.194)	**<0.001**
No. of dissected lymph nodes, <16/≥16	777/647	0.829 (0.734–0.937)	**0.003**	542/408	0.736 (0.630–0.860)	**<0.001**
N stage, 1/2/3	776/429/219	1.428 (1.319–1.546)	**<0.001**	568/256/126	1.449 (1.310–1.603)	**<0.001**
NLN stage, 1/2/3	505/361/558	0.790 (0.737–0.848)	**<0.001**	326/251/373	0.746 (0.682–0.815)	**<0.001**
R_NP,_ stage1/2/3	554/411/459	1.444 (1.343–1.552)	**<0.001**	398/301/251	1.547 (1.407–1.700)	**<0.001**

CI, confidence interval; G1, well differentiated; G2, moderately differentiated; G3, poorly differentiated/undifferentiated; HR, hazard ratio; NLN, negative lymph node; R_NP_, the ratio between negative and positive lymph nodes.

### Univariate survival analysis

**Figure 1:**
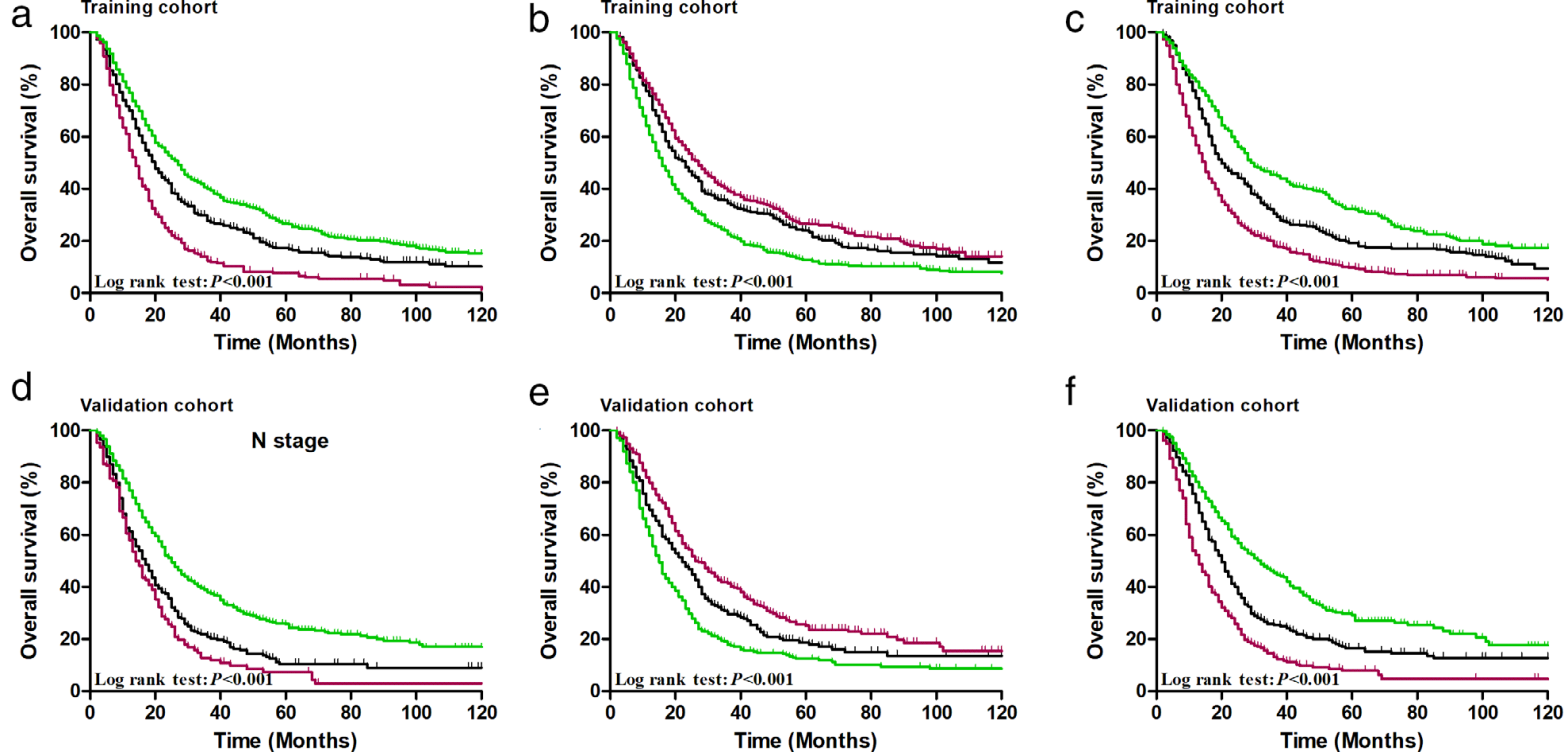
Cumulative five‐year overall survival (OS) curves for esophageal cancer patients stratified by (**a**) N stage 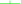, pN1; 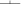, pN2; 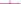, pN3, (**b**) NLN stage 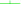, NLN1; 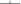, NLN2; 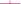, NLN3, (**c**) R_NP_ stage of the training cohort 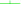, R_NP_1; 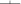, R_NP_2; 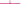, R_NP_3, (**d**) N stage 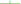, pN1; 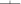, pN2; 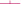, pN3, (**e**) NLN stage 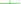, NLN1; 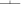, NLN2; 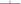, NLN3, (**f**) R_NP_ stage of the validation cohort 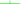, R_NP_1; 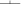, R_NP_2; 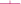, R_NP_3.

The overall survival curves of the two cohorts by N, NLN, and R_NP_ categories are presented in Fig 1. For patients in the training cohort, the five‐year survival rates of N1, N2, and N3 groups under the AJCC nodal staging guidelines were 26.5%, 17.0% and 7.8%, respectively (*P* < 0.001, Fig 1a). The five‐year OS rates for the NLN1, NLN2, and NLN3 groups were 12.7%, 23.9%, and 26.3%, respectively (*P* < 0.001, Fig 1b). For the R_NP_1, R_NP_2, and R_NP_3 groups, the observed five‐year OS rates were 32.0%, 19.0%, and 9.8%, respectively (*P* < 0.001, Fig 1c). In addition, for patients in the validation cohort, the five‐year OS rates of N1, N2, and N3 groups were 26.0%, 10.4% and 7.3%, respectively (*P* < 0.001, Fig 1d). The five‐year OS rates for the NLN1, NLN2, and NLN3 groups were 12.4%, 18.7%, and 25.7%, respectively (*P* < 0.001, Fig 1e). For the R_NP_1, R_NP_2, and R_NP_3 groups, the observed five‐year OS rates were 29.6%, 16.5%, and 7.9%, respectively (*P* < 0.001, Fig 1f).

The clinicopathological characteristics of the two cohorts and the impact of prognostic factors are summarized in Table 1. Intriguingly, significant risk factors found in the training group and further confirmed in the validation cohort were age (both *P* < 0.001), tumor size (*P* < 0.001 and *P* = 0.001, respectively), histological grade (both *P* < 0.001), T stage (both *P* < 0.001), number of dissected lymph nodes (*P =* 0.003 and *P <* 0.001, respectively), N stage (both *P* < 0.001), NLN stage (both *P* < 0.001) and R_NP_ stage (both *P* < 0.001).

### Multivariate survival analysis

The effect of the prognostic variables on survival are described in Table 2. We compared three different lymph node stages, and measured their relationship with EC patient survival. The N stage, contained in Model 1, were statistically significantly related to OS in both the training and validation cohorts. The Model 2 incorporated N stage and NLN stage. While replacing the NLN stage, N and R_NP_ satge were included in the Model 3 to see the difference. We then combined these variables to build the fourth model. In Model 2, N stage and NLN count were statistically significantly related to OS in both cohorts. In Model 3 of the training and validation cohorts for OS, R_NP_ (hazard ratio (HR) = 1.276, 95% confidence interval (CI): 1.137–1.432, *P* < 0.001 and HR = 1.325, 95% CI: 1.146–1.532, *P* < 0.001,respectively) were identified as significant predictors, while N stage (*P* = 0.054 and *P* = 0.058, respectively) was not identified as a significant predictor in the two cohorts. Furthermore, in Model 4, we found that the R_NP_ was correlated with survival (HR = 1.231, 95% CI: 1.072–1.414, *P* = 0.003 and HR = 1.222, 95% CI: 1.029–1.452, *P* = 0.022), but NLN no longer predicted OS (*P* = 0.360 and *P* = 0.091). Other significant prognostic predictors of OS remained as independent factors and included age, histological grade, T stage and N stage (Table 2).

**Table 2:** Multivariate analysis of prognostic factors influencing the survival of esophageal cancer patients with lymph node metastasis

Variables	Training cohort			Validation cohort		
	HR	95% CI	*P*‐value	HR	95% CI	*P*‐value
Model 1						
Age	1.353	1.199–1.527	**<0.001**	1.464	1.253–1.711	**<0.001**
Tumor size	1.041	0.916–1.183	0.537	1.121	0.959–1.312	0.152
Histological grade	1.266	1.131–1.417	**<0.001**	1.293	1.120–1.494	**<0.001**
Dissected lymph nodes	0.766	0.678–0.867	**<0.001**	0.688	0.587–0.806	**<0.001**
T stage	1.137	1.091–1.184	**<0.001**	1.083	1.029–1.139	**0.002**
N stage	1.376	1.266–1.494	**<0.001**	1.422	1.276–1.584	**<0.001**
Model 2						
Age	1.345	1.192–1.517	**<0.001**	1.483	1.269–1.733	**<0.001**
Tumor size	1.050	0.924–1.194	0.450	1.143	0.976–1.338	0.097
Histological grade	1.255	1.121–1.406	**<0.001**	1.266	1.096–1.464	**0.001**
Dissected lymph nodes	0.993	0.807–1.222	0.949	1.010	0.770–1.325	0.942
T stage	1.135	1.090–1.182	**<0.001**	1.085	1.031–1.141	**0.002**
N stage	1.296	1.183–1.420	**<0.001**	1.334	1.190–1.495	**<0.001**
NLN stage	0.831	0.738–0.936	**0.002**	0.763	0.655–0.890	**0.001**
Model 3						
Age	1.352	1.199–1.526	**<0.001**	1.464	1.253–1.711	**<0.001**
Tumor size	1.057	0.930–1.201	0.398	1.121	0.958–1.312	0.155
Histological grade	1.255	1.121–1.406	**<0.001**	1.285	1.113–1.485	**0.001**
Dissected lymph nodes	0.912	0.785–1.059	0.227	0.847	0.697–1.028	0.092
T stage	1.135	1.090–1.182	**<0.001**	1.080	1.027–1.137	**0.003**
N stage	1.131	1.000–1.281	0.054	1.158	0.995–1.347	0.058
R_NP_ stage	1.276	1.137–1.432	**<0.001**	1.325	1.146–1.532	**<0.001**
Model 4						
Age	1.349	1.196–1.523	**<0.001**	1.474	1.261–1.723	**<0.001**
Tumor size	1.058	0.931–1.203	0.388	1.134	0.969–1.328	0.118
Histological grade	1.253	1.119–1.404	**<0.001**	1.272	1.100–1.471	**0.001**
Dissected lymph nodes	0.976	0.791–1.205	0.824	0.998	0.759–1.311	0.986
T stage	1.135	1.090–1.182	**<0.001**	1.082	1.029–1.139	**0.002**
N stage	1.140	1.006–1.292	**0.040**	1.184	1.015–1.381	**0.031**
NLN stage	0.935	0.811–1.079	0.360	0.855	0.731–1.026	0.091
R_NP_ stage	1.231	1.072–1.414	**0.003**	1.222	1.029–1.452	**0.022**

CI, confidence interval; HR, hazard ratio; NLN, negative lymph node; R_NP_, ratio between negative and positive lymph nodes.

### Prognostic prediction accuracy of the various categories of lymph node metastasis

To verify the prognostic performance of the R_NP_ stage on the OS of patients, we performed stratification analyses of the prognostic effect of the R_NP_ classifications on the basis of the different N stages and the number of dissected lymph nodes.

**Figure 2:**
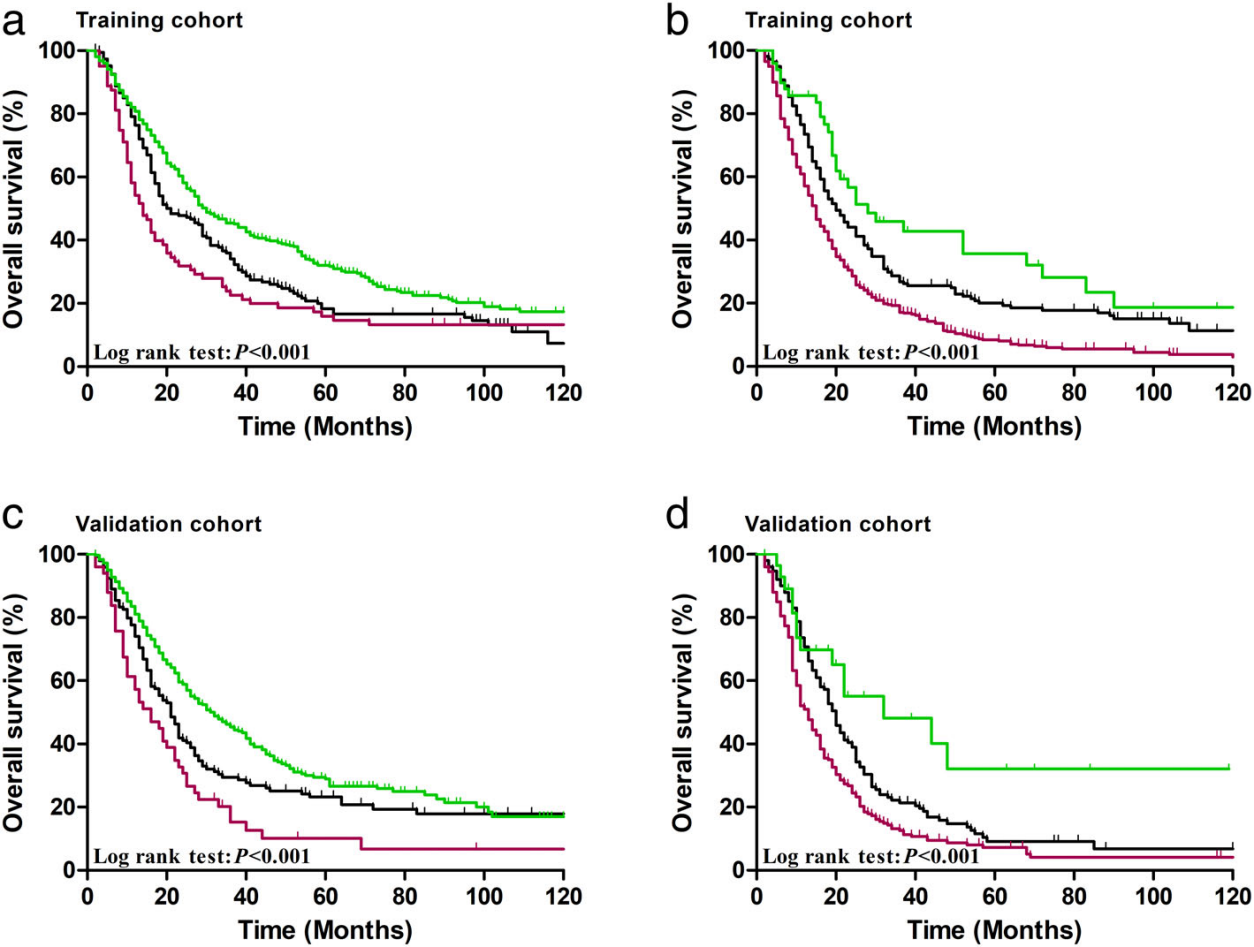
Cumulative five‐year overall survival (OS) curves for ratio between negative and positive lymph nodes (R_NP_) stage in patients with (**a**) N1 stage 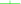, R_NP_1; 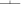, R_NP_2; 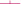, R_NP_3, (**b**) N2 + N3 stage of the training cohort 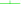, R_NP_1; 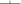, R_NP_2; 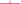, R_NP_3; (**c**) N1 stage 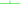, R_NP_1; 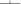, R_NP_2; 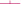, R_NP_3, (**d**) N2 + N3 stage of the validation cohort 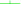, R_NP_1; 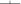, R_NP_2; 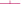, R_NP_3.

In N1 patients of both cohorts, R_NP_ staging was identified as a significant predictor (both *P* < 0.001, Fig 2a and c **)**. In the subgroup which incorporated both N2 and N3 patients, R_NP_ staging was significantly correlated with OS in both training (*P* < 0.001, Fig 2b) and validation cohorts (*P* < 0.001, Fig 2d).

**Figure 3:**
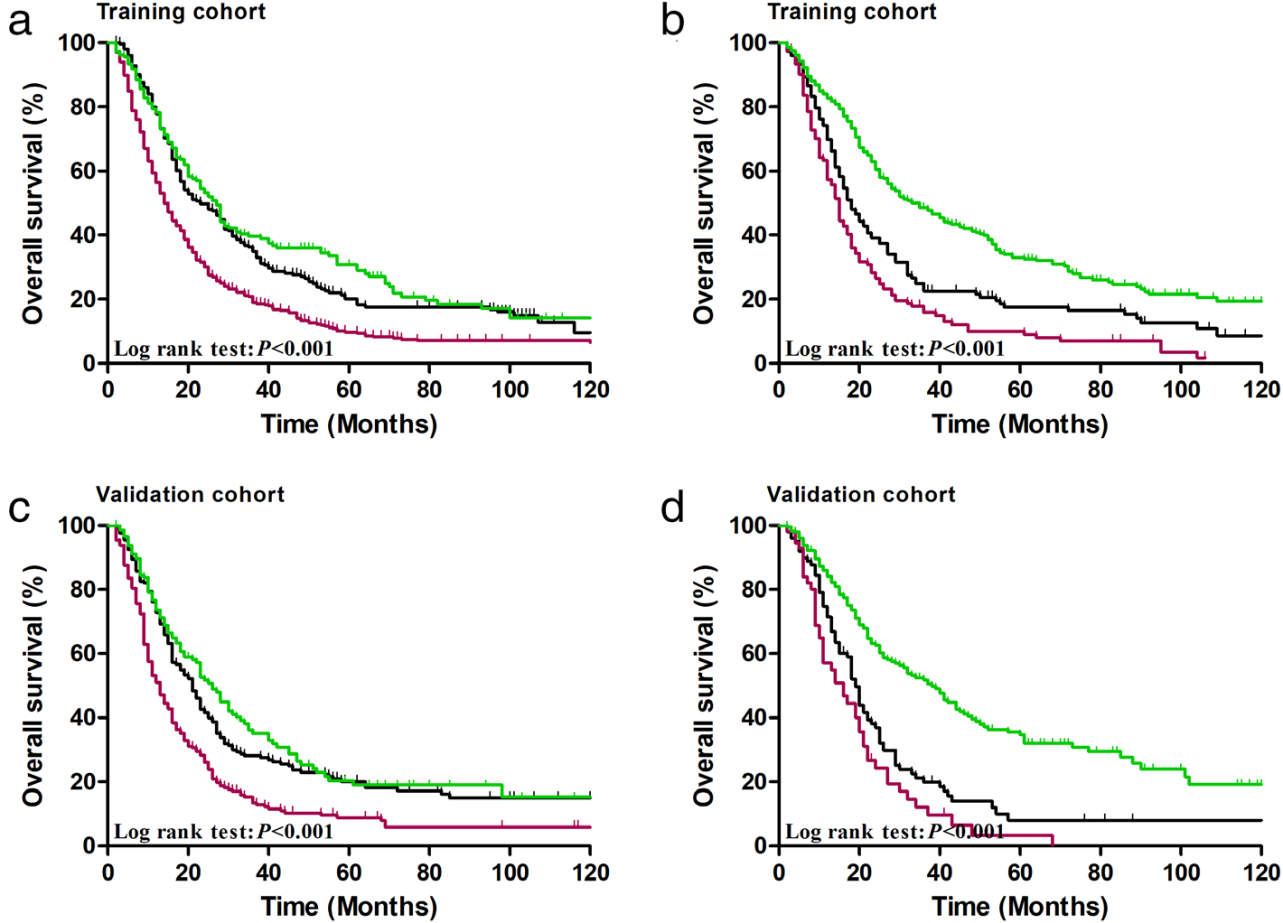
Cumulative five‐year overall survival (OS) curves for ratio between negative and positive lymph nodes (R_NP_) stage in patients with (**a**) dissected lymph nodes <16 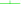, R_NP_1; 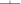, R_NP_2; 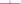, R_NP_3, (**b**) dissected lymph nodes ≥16 of the training cohort 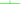, R_NP_1; 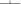, R_NP_2; 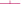, R_NP_3; (**c**) dissected lymph nodes <16 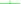, R_NP_1; 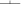, R_NP_2; 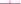, R_NP_3, (**d**) dissected lymph nodes ≥16 of the validation cohort 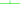, R_NP_1; 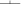, R_NP_2; 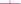, R_NP_3.

We also investigated the prognostic value of the R_NP_ stage on OS in the context of the number of dissected lymph nodes. Figure 3 shows that effect of R_NP_ classifications significantly differed across any number of dissected lymph nodes group in both the training and validation cohorts (both *P* < 0.001) (Fig 3a–d).

### Comparison of the prognostic value between TNM and TR_NP_M classifications

Furthermore, the factor of R_NP_ was incorporated into TNM staging system for EC patients. The two staging systems were directly compared for convenience. With the TNM staging system in the training cohort, 127 cases were stage IIB, 163 cases were stage IIIA, 883 cases were stage IIIB, and 251 cases were stage IVA. Furthermore, 91 cases were stage IIB, 123 cases were stage IIIA, 594 cases were stage IIIB, and 142 cases were stage IVA in the validation cohort. The five‐year OS rates of stage IIB, IIIA, IIIB and IVA EC patients were 43.0%, 33.6%, 18.6% and 8.2%, respectively in the training cohort, while they were 45.6%, 29.9%, 15.3%, 8.6%, respectively in the validation cohort (Table 3).

**Table 3:** Distribution and stage specific survival rates of different classifications for prediction the prognosis of esophageal cancer patients

Variable	Training cohort				Validation cohort			
	Numbers	5‐YSR (%)	HR (95% CI)	*P*‐value	Numbers	5‐YSR (%)	HR (95% CI)	*P*‐value
TNM stage			1.514 (1.396–1.643)	**<0.001**			1.468 (1.325–1.627)	**<0.001**
IIB	127	43.0			91	45.6		
IIIA	163	33.6			123	29.9		
IIIB	883	18.6			594	15.3		
IVA	251	8.2			142	8.6		
TR_Np_M stage							1.608 (1.451–1.782)	**<0.001**
IIB	81	48.5	1.531 (1.414–1.656)	**<0.001**	67	52.1		
IIIA	164	38.2			109	34.8		
IIIB	687	21.0			505	17.9		
IVA	492	10.4			269	8.4		

5‐YSR, five‐year survival rate; CI, confidence interval; HR, hazard ratio; TNM, tumor‐node‐metastasis; TR_NP_M, tumor‐R_NP_‐metastasis.

**Figure 4:**
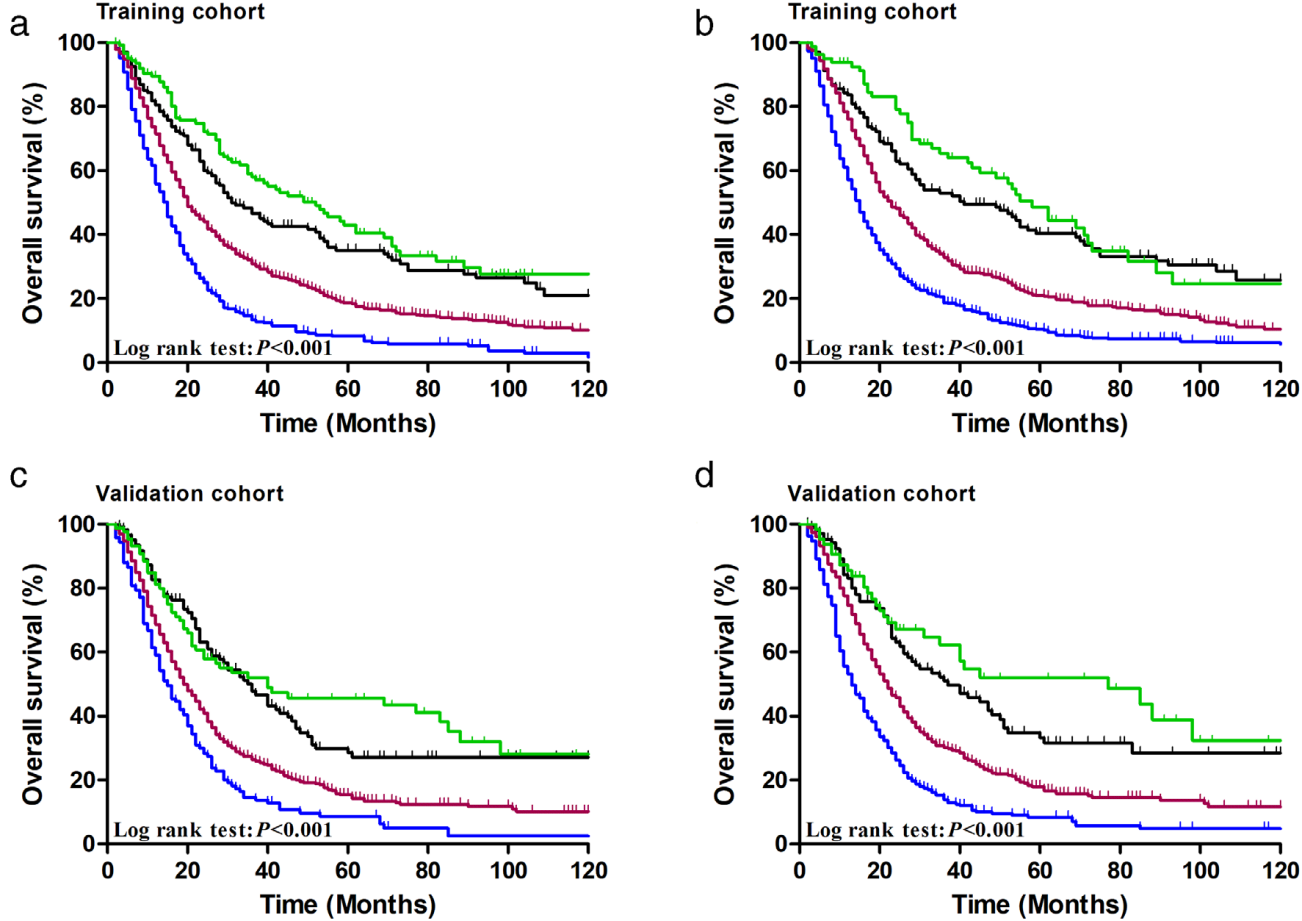
Cumulative five‐year overall survival (OS) curves for esophageal cancer patients according to (**a**) the TNM staging system 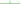, IIB; 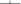, IIIA; 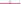, IIIB; 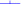, IVA, (**b**) TR_NP_M staging system of the training cohort 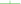, IIB; 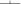, IIIA; 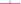, IIIB; 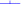, IVA; (**c**) TNM staging system 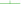, IIB; 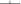, IIIA; 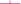, IIIB; 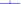, IVA, (**d**) TR_NP_M staging system of the validation cohort 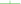, IIB; 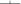, IIIA; 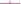, IIIB; 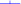, IVA.

With the TR_NP_M staging system, there were 81 stage IIB patients, 164 stage IIIA patients, 687 stage IIIB patients, and 492 stage IVA patients in the training cohort. Furthermore, 67 cases were stage IIB, 109 cases were stage IIIA, 505 cases were stage IIIB, and 269 cases were stage IVA in the validation cohort. The five‐year OS rates of stage IIB, IIIA, IIIB and IVA patients were 48.5%, 38.2%, 21.0% and 10.4%, respectively, while they were 52.1%, 34.8%, 17.9% and 8.4%, respectively in the validation cohort. Therefore, the TR_NP_M staging system had a greater statistical significance comparable to the TNM staging system in both independent cohorts (*P* < 0.001, respectively) (Fig 4a‐d).

### Comparison of the prognostic superiority between N, R_NP_, TNM and TR_NP_M classifications

We used three parameters to compare the N and R_NP_ classification; linear trend χ^2^ score, likelihood ratio χ^2^ score and AIC value. The higher linear trend χ^2^ score and higher likelihood ratio χ^2^ score, the better the system, whereas the lower the AIC value, the better the system. In the multivariable regression analyses, they were all independent factors of overall survival (both *P* < 0.001). We found that the linear trend χ^2^ scores were 55.24 and 95.42 of N and R_NP_, respectively in the training cohort, while they were 30.51 and 69.50 in the validation cohort. While the likelihood ratio χ^2^ scores were 72.88 and 99.32 of N and R_NP_, respectively in the training cohort, they were 48.27 and 80.84 in the validation cohort. From the results of the AIC value in Cox regression, we determined that the AIC of R_NP_ was lower than that of N stage (Table 4). Therefore, we considered that R_NP_ had the better discrimination ability for obvious improvement in the accuracy of prognostic prediction for EC patients than the N classification.

**Table 4:** Comparison of the prognostic performance of different staging systems

Classification	Subgroups	Training cohort				Validation cohort			
		Figure	Linear trend χ^2^	Likelihood ratio χ^2^	AIC	Figure	Linear trend χ^2^	Likelihood ratio χ^2^	AIC
N stage	N1, N2, N3	1A	55.24	72.88	13 831.30	1D	30.51	48.27	8093.79
R_NP_ stage	R_NP_ 1, R_NP_2, R_NP_3	1C	95.42	99.32	13 804.86	1F	69.50	80.84	8061.22
TNM stage	IIB, IIIA, IIIB, IIIC	4A	72.09	108.48	13 795.70	4C	34.28	57.88	8084.18
TR_NP_M stage	IIB, IIIA, IIIB, IIIC	4B	100.36	122.91	13 781.27	4D	66.54	90.10	8051.96

AIC, Akaike information criterion; R_NP_, ratio between negative and positive lymph nodes; TNM, tumor‐node‐metastasis; TR_NP_M, tumor‐R_NP_‐metastasis.

The linear trend χ^2^ scores, likelihood ratio χ^2^ scores, and AIC values were also used to compare the prognostic performance of the two staging systems. We found that the TR_NP_M classification had the higher linear trend χ^2^ scores, likelihood ratio χ^2^ scores and lower AIC values compared to the TNM staging system in both cohorts (Table 4). We therefore demonstrated that the performance of the TR_NP_M staging system is superior to the traditional TNM staging system in predicting the survival of EC patients after esophagectomy.

### Prognostic nomograms for predicting the survival of EC patients

**Figure 5:**
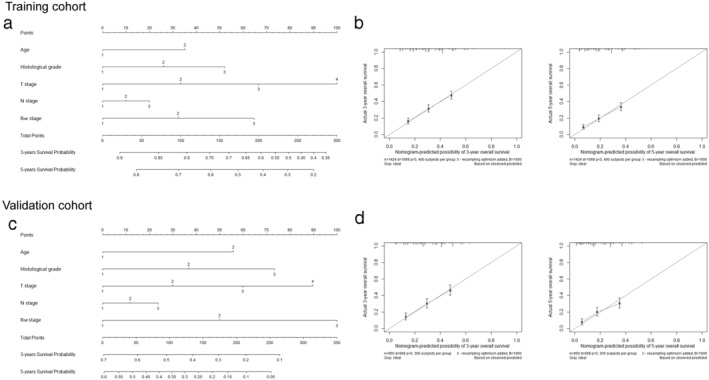
Nomogram for predicting cumulative five‐year overall survival (OS) in esophageal cancer patients. The sum of the points assigned to each factor by the nomogram is located on the total point axis, and a line is drawn downward to the survival axes to determine the probability of five‐year OS. The c‐indexes values for the training cohort (**a**) and the validation cohort (**c**) are 0.648 and 0.674, respectively. Calibration curves for predicting three‐ and five‐year OS, which are indicative of predictive accuracy, for the training cohort (**b**) and the validation cohort (**d**). The x‐axis represents the nomogram‐predicted survival, and the actual survival is plotted on the y‐axis. The dotted line represents the ideal correlation between predicted and actual survival.

Furthermore, nomograms were used to calculate the three‐ and five‐year OS of patients. R_NP_ was selected as an independent prognostic predictor in nomograms in both training and validation cohorts, which were identical to those in the aforementioned multivariate analyses conducted by Cox regression. In the training group, the C‐index for predicting OS with the formulated nomogram was 0.648. The calibration curves exhibited optimal consistency between the actual observation of OS and nomogram‐predicted OS at three‐ and five‐years after surgery (Fig 5a and c). In the validation cohort, the C‐index for OS prediction was 0.674. The calibration plot in such group for OS prediction at three‐ and five‐years also fitted very well between the observation and the prediction nomogram (Fig 5b and d).

## Discussion

Our study analyzed the OS of two random cohorts of EC patients who underwent radical surgery and assessed the prognostic prediction performance of N, NLN and R_NP_. We confirmed R_NP_ acted as significant prognostic factor in both the training and validation cohorts. Our nomogram also confirmed the prognostic significance of the R_NP_ staging system in EC patients.

Lymph node status is considered one of the key elements which influence the treatment decision of esophageal cancer patients. To the best of our knowledge, the N category and NLNs are identified by the number of PLNs. Thus, an inadequate number of dissected lymph nodes will influence lymph node count, further affecting treatment and prognosis.^17^, ^18^, ^19^ To accurately assess lymph node metastasis for improving long‐term outcomes, previous studies have included several different prognostic factors such as N, positive lymph node ratio, log odds of positive lymph nodes and NLN.^2^, ^9^, ^13^, ^19^, ^20^, ^21^, ^22^ However, controversies still exist over which lymph node metastasis factor system is optimal for accurately predicting patient prognosis following radical esophagectomy.

To help eliminate the effect of the number of lymph nodes dissected on N and NLN count, we propose R_NP_ as a new prognostic indicator. In recent years, R_NP_ has attracted attention in gastric cancer and colon cancer as a novel category of lymph node metastasis. In the two studies by Deng *et al*.^14^, ^23^ it was demonstrated that R_NP_ could help improve the accuracy of prognostic evaluation when compared with other prognostic factors, and was recommended for use in predicting OS of GC patients. To date, little research has been devoted to elucidating the prognostic value of R_NP_ in EC patients.

The univariate analysis demonstrated that the three lymph node categories, including N, NLN and R_NP_ stages, were all significantly associated with survival. We further conducted multivariate analyses and established four models. After eliminating the influence of confounders, we found the R_NP_ remained statistically significant among all the established models, whereas the N and NLN stage were not significant in Model 3 and Model 4, respectively. To further verify the prognostic performance of OS in EC patients, we performed a log‐rank test on the three matched R_NP_ subgroups on the basis of the different N stages and the number of dissected lymph nodes. Stratification analysis of the training cohort identified R_NP_ as appropriate for distinguishing evaluation survival differences for all N subgroup patients. As for the validation cohort, R_NP_ was identified as applicable for distinguishing evaluation survival differences between patients of N stage and patients with fewer or more than 16 dissected lymph nodes. Lower R_NP_ stage was also associated with better survival regardless of the number of dissected lymph nodes in both cohorts. Therefore, we deduced R_NP_ could serve as the optimal category for EC patients who underwent radical surgery.

We found that R_NP_ had higher linear trend χ^2^ score, higher likelihood ratio χ^2^ score and smaller AIC value in Cox regression than the N stage, which implied that R_NP_ had the better ability to exactly predict the prognosis of patients. The results of the validation cohort were consistent with the training cohort. Furthermore, our novel TR_NP_M staging system, which uses R_NP_ instead of N staging, demonstrated better discrimination in EC patients compared to the TNM staging system according to the higher linear trend χ^2^ score, higher likelihood ratio χ^2^ score and smaller AIC value in both cohorts. Thus, the TR_NP_M staging system is more reliable for exact evaluation of the prognosis for patients than TNM staging system. Thus, we suggest that the R_NP_ staging system can be used as a novel factor describing lymph node metastasis for predicting the prognosis of EC patients. In the current study, we constructed nomograms to predict survival in two cohort EC patients. The nomogram accurately predicted three‐, and five‐year overall survival in the training and validation cohorts; C‐indexes confirmed the accuracy of these predictions and the calibration curves confirmed the predictive ability.

There are several limitations of our study that should be addressed. First, the survival dataset is incomplete and cannot be completed since this study was a retrospective analysis. To address this in the future, prospective studies are needed to confirm our results. Second, since the ratio of negative to positive lymph nodes was calculated, we excluded patients without positive lymph nodes. Therefore, only patients with specific lymph node metastases were involved in the analysis. Third, the SEER database was used for this study. Although this database is large with extensive long‐term follow‐up information, it lacks data correlated with survival, including adjuvant treatments, comorbidities and chemotherapy regimens and dosage. Also, whether using adjuvant therapy or not has an inevitable impact on surgical treatments for survival, especially for node‐positive EC patients, remains to be determined. Therefore, the broader applicability of our results may be limited. In addition, the lack of detailed treatment information may have biased the results of the study.

In conclusion, the results of this study confirmed that R_NP_ is more accurate than the N staging system in predicting survival and reflects comprehensive information on lymph node dissection and positive and negative lymph node count. R_NP_ can be used as a valuable indicator to provide prognostic guidance for lymph node positive EC patients. The novel TR_NP_M staging system based on R_NP_ should be considered as an alternative to the current TNM classification.

## Disclosure

No potential conflicts of interest are disclosed.
